# Pure Ion Chromatograms Combined with Advanced Machine Learning Methods Improve Accuracy of Discriminant Models in LC–MS-Based Untargeted Metabolomics

**DOI:** 10.3390/molecules26092715

**Published:** 2021-05-05

**Authors:** Miao Tian, Zhonglong Lin, Xu Wang, Jing Yang, Wentao Zhao, Hongmei Lu, Zhimin Zhang, Yi Chen

**Affiliations:** 1 College of Chemistry and Chemical Engineering, Central South University, Changsha 410083, China; miaolcq@csu.edu.cn (M.T.); hongmeilu@csu.edu.cn (H.L.); 2 Yunnan Academy of Tobacco Agricultural Sciences, Kunming 650021, China; sdlzl1983@163.com; 3 Shanghai New Tobacco Product Research Institute Limited Company, Shanghai 200082, China; wangx@sh.tobacco.com.cn (X.W.); yzc1985257@163.com (J.Y.); gj201323050@163.com (W.Z.)

**Keywords:** Pure Ion Chromatogram, UMAP, XGBoost, KPIC2, LC–MS

## Abstract

Untargeted metabolomics based on liquid chromatography coupled with mass spectrometry (LC–MS) can detect thousands of features in samples and produce highly complex datasets. The accurate extraction of meaningful features and the building of discriminant models are two crucial steps in the data analysis pipeline of untargeted metabolomics. In this study, pure ion chromatograms were extracted from a liquor dataset and left-sided colon cancer (LCC) dataset by K-means-clustering-based Pure Ion Chromatogram extraction method version 2.0 (KPIC2). Then, the nonlinear low-dimensional embedding by uniform manifold approximation and projection (UMAP) showed the separation of samples from different groups in reduced dimensions. The discriminant models were established by extreme gradient boosting (XGBoost) based on the features extracted by KPIC2. Results showed that features extracted by KPIC2 achieved 100% classification accuracy on the test sets of the liquor dataset and the LCC dataset, which demonstrated the rationality of the XGBoost model based on KPIC2 compared with the results of XCMS (92% and 96% for liquor and LCC datasets respectively). Finally, XGBoost can achieve better performance than the linear method and traditional nonlinear modeling methods on these datasets. UMAP and XGBoost are integrated into KPIC2 package to extend its performance in complex situations, which are not only able to effectively process nonlinear dataset but also can greatly improve the accuracy of data analysis in non-target metabolomics.

## 1. Introduction

Metabolomics aims at the unbiased and comprehensive quantification of metabolites in organisms, tissues, or cells [1,2]. For untargeted metabolomics, its goal is the simultaneous detection of as many metabolites as possible in samples and the discovery of metabolomic changes between groups [3]. At present, the combination of chromatography and mass spectrometry has become the key technology for the analysis of metabolites in biological systems [4,5]. Compaed with gas chromatography coupled to mass spectrometry (GC–MS) [6,7,8], high-performance liquid chromatography–mass spectrometry (LC–MS) can analyze compounds with semi-polar and lower volatility in a wider mass range without derivatization [9,10,11]. The samples are separated by the chromatographic column after injection and identified by analyzing the spectra acquired by the mass spectrometer. Since each eluted metabolite produces multiple mass signals, such as fragments, adducts and isotope peaks, LC–MS data contain thousands of metabolomic features for complex samples. Therefore, the pre-processing methods are needed to extract meaningful features for further statistical analysis.

The data preprocessing methods should extract the features in each sample, align the features between samples, and obtain the table containing *m/z*, retention time, and intensity. Ideally, the extracted feature of one metabolite should not contain the information from other metabolites with similar retention time and *m/z*. Many software tools have been developed to automate the preprocessing of LC–MS datasets, such as MetAlign [12,13], MZmine [14,15], XCMS [16,17] and OpenMS [18,19,20]. Traditional methods split *m/z* axis into bins, construct the extracted ion chromatogram (EIC) and detect the features in EICs. This strategy was widely used in preprocessing of LC–MS datasets. However, the ions of the same metabolite are sometimes assigned to two adjacent *m/z* bins. To solve this problem, the matchedFilter algorithm was integrated into XCMS, which creates overlapping combined chromatograms for peak detection [16]. However, it is difficult to handle the co-eluting peaks with similar *m/z* in the same bin. The centroidPicker in MZmine has the same problem as well [15]. Therefore, the centWave method was proposed, which combined the regions of interest and the continuous wavelet transform (CWT)-based approach for chromatographic peak deconvolution [21]. The centWave method avoids disadvantages of binning to some extent, and multi-scale peak detection can achieve high sensitivity, so it has been quickly integrated into XCMS and MZmine. To this day, it still one of the most popular feature extraction methods in LC–MS analysis. However, the relative mass difference between adjacent ions is related to intensity, and the relative mass difference tolerance of different mass spectrometers is also different [22]. Moreover, the change in temperature will affect the high voltage drift of the power during heating, which will affect the quality and accuracy of the time of flight (TOF) instruments [23]. Therefore, the fixed tolerance may split peaks due to the inconsistent relative mass differences [24].

To avoid the mentioned shortcomings of the previous methods, the pure ion chromatogram (PIC) has been proposed [21,25]. It is particularly suitable for high-resolution LC–MS datasets. PIC refers to the chromatogram containing only one intensity per scan, and the intensity should come from the ions derived from the same metabolite. There are many PIC-based methods, including TracMass [25], Massifquant [26], TracMass2 [27] and PITracer [24]. The TracMass method tracks data points in *m/z* and intensity space by Kalman tracking. It can extract PICs without noises and eliminate the problems related to binning. However, the source code of TracMass is not publicly available. Subsequently, Massifquant was implemented and open-sourced. Since the Kalman filter is complex and time-consuming, the pure ion is tracked by the greedy nearest neighbor strategy in TracMass2. It assigns a PIC ID to each data point in the first scan. In the next scan, the data points are assigned the same PIC ID if the *m*/*z* value is close enough to the PIC of the previous scan. If there is no PIC with a similar *m*/*z* value in the previous scan, a new PIC ID is assigned to the data point. TracMass2 can get the same results as TracMass with much faster speed. PITracer can adaptively estimate the relative mass difference tolerance for each scan and calibrate the *m/z* value to remove discontinuous ions in LC–MS profiles. However, if there are missing points and irregular distribution in a scan, the length of the PIC will be shortened and separated. In LC–MS datasets, the ions of the same metabolite have almost the same *m/z* values, and these ions tend to cluster in the *m/z* dimension. KPIC extracts PIC by k-means clustering to avoid the above problems [28]. Then, an integrated framework called K-means-clustering-based Pure Ion Chromatogram extraction method version 2.0 (KPIC2) was developed, which improves the accuracy of PIC extraction by considering the intensity of ions [29]. In addition, KPIC2 includes peak detection, peak alignment, grouping, missing value filling, and traditional pattern recognition for analyzing LC–MS-based metabolomic datasets.

After the construction of the peak table, it is important to visualize the dataset before further statistical analysis. However, the dimensionality reduction and visualization methods are not provided in KPIC2. Principal component analysis (PCA) is the most widely used dimensionality reduction method through the linear combination of the original variables [30,31,32]. However, PCA tries to preserve the global structure of the data at the risk of losing the local structure, and it may fail on the nonlinear and complex dataset. The t-distributed stochastic neighbor embedding (t-SNE) is a non-linear dimensionality reduction method that converts the similarity of data points into joint probabilities and minimizing the Kullback–Leibler divergence between low-dimensional data and high-dimensional data [33]. However, t-SNE suffers from limitations such as loss of global data structure, slow calculation speed and inability to meaningfully represent big datasets [34]. The unified manifold approximation and projection (UMAP) has been constructed from a theoretical framework based on Riemannian geometry and algebraic topology [35,36]. It can achieve a better balance between the local data structures and global data structures than t-SNE and PCA.

The discriminant models should be built to learn the decision rules, predict new samples and screen the biomarkers. Therefore, advanced machine learning methods and variable selection methods are required. Partial least squares discriminant analysis (PLS-DA) [37], orthogonal partial least squares discriminant analysis (OPLS-DA) [38] and Random Forest (RF) [39] were implemented in KPIC2 for pattern recognition and biomarker selection. PLS–DA is widely used in chemometrics and metabolomics, and it is based on the group membership encoding and the partial least squares regression [40]. OPLS–DA [41] divides the variables into predictive and orthogonal information with orthogonal signal correction (OSC) technology [42]. Compared with PLS–DA, it provides better visualization and interpretation and has been widely used in modeling and biomarker discovery for metabolomics [43,44]. However, PLS–DA and OPLS–DA are suitable for linear and collinearity datasets and may fail on the nonlinear datasets. RF is an ensemble machine learning method based on classification and regression tree (CART), and it can be used to classify high-dimensional and nonlinear datasets well [39]. In 2014, extreme gradient boosting (XGBoost) was released by Chen et al., which can achieve the goal of fast calculation and excellent performance [45,46]. It builds multiple weak learners on the bootstrapped data, trains the subsequent models, and aggregates the models to reduce both the variance and bias of the prediction.

**Scheme 1:**
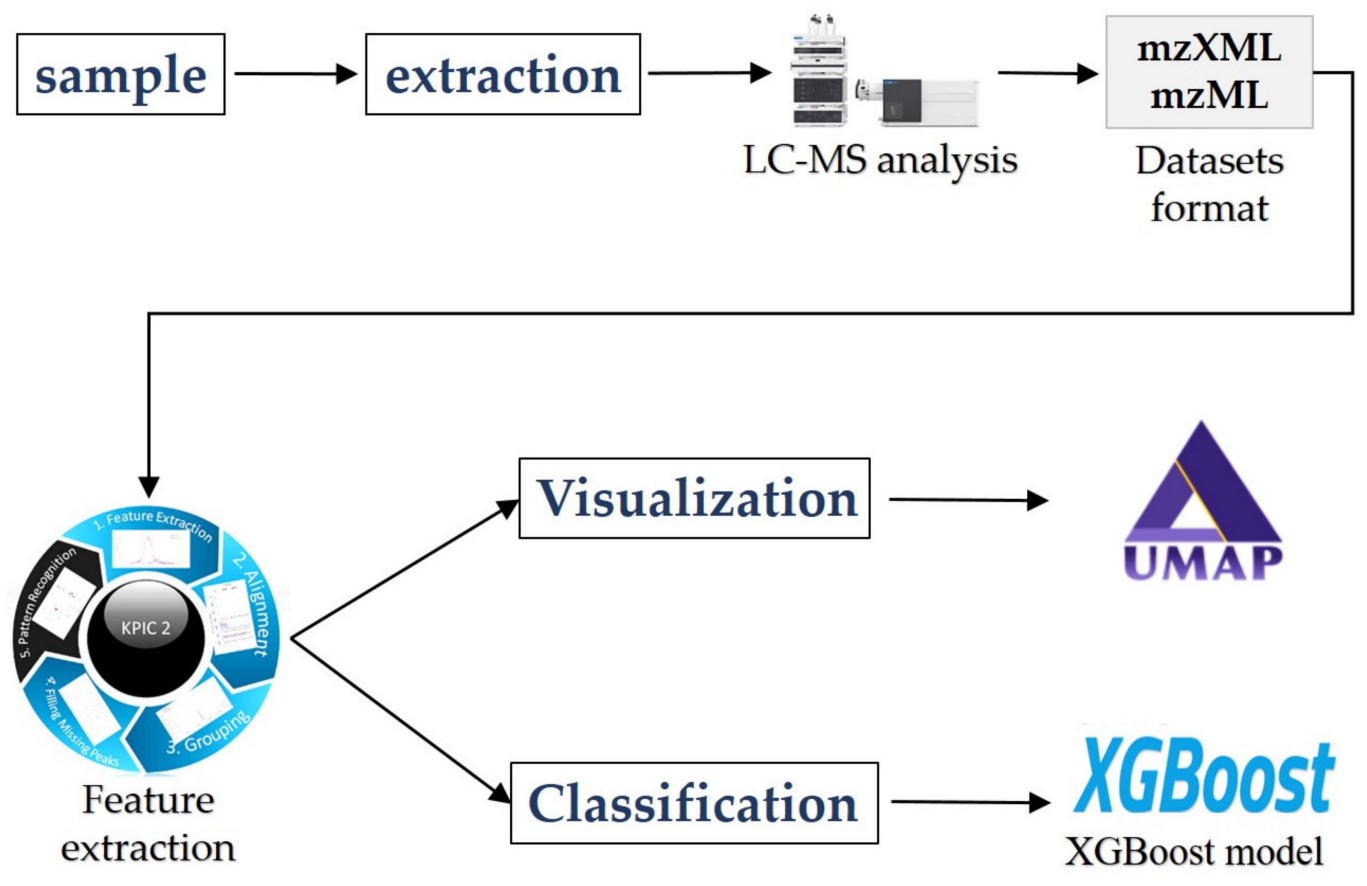
Schematic diagram of the proposed data analysis pipeline for complex liquid chromatography-mass spectrometry (LC–MS)-based untargeted metabolomics. It can be divided into five parts: the extraction of metabolites from samples, LC–MS analysis, data preprocessing, visualization and statistical analysis. In the study, K-means-clustering-based Pure Ion Chromatogram extraction method version 2.0 (KPIC2) is used to extract pure ion chromatograms. The samples from different groups are visualized through uniform manifold approximation and projection (UMAP). Extreme gradient boosting (XGBoost) is used to build discriminant models and screen differential metabolites.

In this study, advanced machine learning methods, UMAP and XGBoost, are integrated into KPIC2 to improve its performances on complex LC–MS-based metabolomic datasets. The KPIC2 framework was applied to perform extraction of PICs, peak detection, peak alignment, grouping and missing value filling for the liquor and LCC datasets. Peak tables containing retention time, *m/z* and intensity were obtained. The features were visualized by PCA, t-SNE and UMAP respectively, and XGBoost was used to build the discriminant models. Since XCMS is still widely used, it is necessary to study the performance of the XGBoost model based on KPIC2 compared with XCMS. Therefore, the proposed pipeline comprising KPIC2 and XGBoost was compared with XCMS by evaluating the accuracy of the models. In order to evaluate the advantages of the XGBoost model, the XGBoost model based on the features extracted by KPIC2 was compared with PLS–DA, support vector machine (SVM) and Random Forest (RF). The schematic diagram of this proposed data analysis pipeline is depicted in Scheme 1.

## 2. Materials and Methods

### 2.1. Theory of KPIC2

The details of the theoretical parts of KPIC2 have been described in the reference [29]. KPIC2 is an integrated framework and has been developed for metabolomics studies. It can extract pure ions by the optimal k-means clustering, detect pure ions accurately, align PICs across samples, group PICs to identify isotope and potential adduct PICs, fill missing peaks and perform multivariate pattern recognition. KPIC2 is an effective analytical framework for metabolomics datasets that integrates the concept of pure ion chromatograms, which can improve the accuracy of quantification, classification and biomarker identification. In addition, KPIC2 is implemented in R programming language and can be used as an open-source software package.

### 2.2. Theory of Advanced Machine Learning Methods

#### 2.2.1. Visualization Methods

UMAP is a novel manifold learning technique for dimensionality reduction, which can be used for visualization and non-linear dimensionality reduction [35]. The algorithm is based on three assumptions about the data: (1) the data are uniformly distributed on the Riemannian manifold; (2) the Riemannian metric is locally constant; (3) the manifold is locally connected. Based on the above assumptions, UMAP can model manifolds with fuzzy topology. The embedding is found by searching for the low-dimensional projection that has the closest equivalent fuzzy topology data. Compared with t-SNE and PCA, its dimensionality reduction calculation speed is fast and can achieve a better balance between the local and global data structures. This allows us to generate high-quality embedding of larger datasets in two or three dimensions for visualization.

#### 2.2.2. XGBoost

XGBoost is an optimized and highly efficient gradient boosting decision tree (GBDT) implementation and can solve problems beyond billions of samples [45]. It performs the second-order Taylor expansion of the loss function and adds a regular term, which effectively avoids overfitting and speeds up the convergence. XGBoost continuously creates new decision trees and fits the residuals of previous predictions to improve the accuracy. It can be expressed in a form of addition in Equation (1):

(1) $${\overset{\wedge }{y}}_{i}=\sum_{k=1}^{K}f_{k}(x_{i}),f_{k}\in F$$

Among them, ${\overset{{}_{\wedge }}{y}}_{i}$ is the predicted value of the model. K is the number of trees. $f_{k}$ represents the *k*-th sub-model. $x_{i}$ is the *i*-th sample in data; *F* represents the set of all trees.

First, a number of training samples consisting of input vector **x** and output variable *y* are randomly given $T=(x_{1},y_{1}),(x_{2},y_{2}),\cdots ,(x_{N},y_{N})$. The XGBoost model is trained by optimizing the objective function. The objective function of XGBoost consists of a loss function and a regular term:

(2) $$obj=\sum_{i=1}^{n}l(y_{i},{\overset{\wedge }{y}}_{i^{{}^{{}^{\left(t\right)}}}})+\sum_{i=1}^{t}\Omega (f_{i})$$

(3) $$\Omega (f)=\gamma T+\frac{1}{2}\lambda {\left\|\omega \right\|}^{2}$$

obj is the objective function of the t-th iteration. ${\overset{\wedge }{y}}_{i^{{}^{{}^{\left(t\right)}}}}$ is the predicted value of the *t*-th iteration. $\Omega (f_{k})$ represents the regular term of model of the *t*-th iteration. The γ and λ are the parameters of the regular term to control the complexity of the decision trees. *T* is the number of leaf nodes in the decision tree. The regular term $\Omega (f_{k})$ can simplify models and prevent over-fitting.

Taylor expansion was applied to this objective function. $l(y_{i},{\overset{\wedge }{y}}_{i^{{}^{{}^{\left(t-1\right)}}}})$ is the loss function of the *i*-th samples with ${\overset{\wedge }{y}}_{i^{{}^{{}^{\left(t-1\right)}}}}$ as the independent variable, and $g_{i}$ and $h_{i}$ are their first and second derivative, respectively. This makes the value of the objective function only depend on $g_{i}$ and $h_{i}$. The minimum of the objective function can be found by the derivation. Then, important variable features are selected by finding the optimal split point, and the scoring formula used for node splitting in the tree model can be derived:

(4) $$Gain=\frac{1}{2}\left[\frac{{\left(\sum {}_{i\in I_{L}}g_{i}\right)}^{2}}{\sum {}_{i\in I_{L}}h_{i}+\lambda }+\frac{{\left(\sum {}_{i\in I_{R}}g_{i}\right)}^{2}}{\sum {}_{i\in I_{R}}h_{i}+\lambda }-\frac{{\left(\sum {}_{i\in I}g_{i}\right)}^{2}}{\sum {}_{i\in I}h_{i}+\lambda }\right]-\gamma$$

Here, I is a subset of the available observations in the current node and $I_{L}$, $I_{R}$ are subsets of the available observations in the left and right nodes after the split. Finally, the XGBoost boosted tree is obtained through successive loop iterations.

There are three reasons XGBoost has better performance and efficiency than GBDT: (1) The regular term is introduced in the XGBoost model to control the complexity of the model, which makes the trained model simpler and prevents overfitting. (2) Before the XGBoost model is trained, the data are sorted in advance and then saved as a block structure. Therefore, the gain calculation of each feature can be performed in multiple threads. (3) Column-sampling works similarly to random forests, which not only reduces overfitting but also reduces calculations.

### 2.3. Datasets

#### 2.3.1. Liquor Dataset

This dataset is publicly available, and it was described in reference [47]. Six types of liquor samples (BJ-1, BJ-2, BJ-3, BJ-4, BJ-5, BJ-6) were purchased from the local market, and 6 repeats were sampled for each type. Each type of liquor was brewed through a special winemaking process. The specific experimental procedure is as follows: 500 μL of liquor sample was added to the sample tube, and the sample was diluted to 1 mL using water/methanol (1/1, v/v) solution. The samples were then vortexed for 5 min and analyzed by Agilent 1290–6545 UPLC-QTOF (Agilent Technologies, Santa Clara, CA, USA).

#### 2.3.2. LCC Dataset

This is also publicly available on the MetaboLights repository with the identifier MTBLS1129. Samples of the left-sided colon cancer (LCC) dataset were obtained from surgical colectomy specimens, and they were selected from male patients who were ≥55 years old. All normal colon tissues were selected from stage I-IV colorectal cancer (CRC) patients (n = 27), and tumor tissue samples were selected from LCC stage I-III (n = 54). The detailed experimental protocol of this dataset was described in reference [48]. A UPLC system (H-Class ACQUITY, Waters Corporation, MA, USA) coupled to a quadrupole time-of-flight (QTOF) mass spectrometer (Xevo G2-XS QTOF, Waters Corporation, MA, USA) was used for MS data acquisition.

### 2.4. Comparison of KPIC2 and XCMS

The raw data of LC–MS instruments are often stored in a proprietary format. Therefore, raw files were exported in mzXML format and converted from mzXML to mzML format using OpenMS (version = 2.4.0, Python package) [19]. The mzML files were imported into XCMS (version = 3.11.3, R package) for data preprocessing. The mzXML files were imported into KPIC2 (version = 2.4.0, R package) for data preprocessing.

KPIC2 is an integrated framework for metabolomics research. It can accurately detect PICs, align PICs between samples, group PICs to identify isotopes and potential adducts, and fill in missing peaks. XCMS is the de facto standard to process untargeted metabolomic data, which comprises nonlinear retention time alignment, matching filtering, peak detection, peak matching and missing value filling. CAMERA [49] (version = 1.44.0, R package) is used to annotate the isotope peaks and adduct ions in peak lists detected by XCMS. KPIC2 and XCMS were applied to the liquor dataset and the LCC dataset. Feature detection, peak alignment, grouping between samples and missing value filling were performed on these datasets. The peak tables containing retention time, *m/z* and intensity were obtained for subsequent statistical analysis.

### 2.5. Pattern Recognition

With the extracted features in the previous section, advanced machine learning methods were applied for pattern recognition of the LC–MS-based metabolomic datasets, including visualization and modeling. The features were normalized by removing the mean and scaling to unit variance. Samples in the liquor dataset and the LCC dataset were dimensionally reduced and visualized through PCA, t-SNE and UMAP. The low-dimensional plots can discover the patterns in the datasets. Then, each dataset was divided randomly into the training set (67%) and test set (33%). The training set was used to build the XGBoost models, and the test set was used to evaluate their performances. In order to establish reliable and accurate models, some important parameters in XGBoost were optimized. In addition, some traditional methods, including PLS-DA, SVM (kernel = “rbf”) and RF, were applied to the classification between different samples in each dataset, and their parameters were optimized through Grid Search. Finally, their classification results were evaluated by evaluation criteria for discriminant models.

### 2.6. Evaluation Criteria

The performance of the XGBoost models must be evaluated by the proper evaluation criteria. In this study, the accuracy, precision, recall, F1 score (F1_score) and receiver operating characteristic (ROC) curve were used as the criteria to evaluate the XGBoost models.

The accuracy is the ratio of the number of samples correctly classified by the classifier to the total number of samples in the dataset, as shown in Equation (5):

(5) $$Accuracy=\frac{TP+TN}{TP+TN+FP+FN}$$

where TP is the number of positive samples predicted to be positive by the model, TN is the number of negative samples predicted to be negative by the model, FP is the number of negative samples predicted to be positive by the model and FN is the number of positive samples predicted to be negative by the model. 

The precision is the ratio of the number of positive samples predicted correctly to the number of positive samples predicted, as shown in Equation (6):

(6) $$Precision=\frac{TP}{TP+FP}$$

The recall (or sensitivity) is the ratio of the number of positive samples predicted correctly to the total number of positive samples, as shown in Equation (7).

(7) $$Recall=\frac{TP}{TP+FN}$$

The specificity is the ratio of the number of negative samples predicted correctly to the total number of negative samples, as shown in Equation (8).

(8) $$Specificity=\frac{TN}{TN+FP}$$

F1_score is the harmonic mean of precision and recall, as shown in Equation (9).

(9) $$F1\_score=\frac{2*Precision*Recall}{Precision+Recall}$$

The ROC curve is a comprehensive criterion that reflects the sensitivity and specificity of continuous variables. For binary classification problems, each point of the curve represents a threshold, and the classifier gives each sample a score. If the score is greater than the threshold, we consider it as a positive sample, and if the score is less than the threshold, we consider it as a negative sample. The horizontal axis of the curve is the false positive rate, that is, the ratio of the number of negative samples predicted error to the total negative samples. The vertical axis of the curve is the true positive rate, that is, the ratio of the number of positive samples predicted correctly to the total number of positive samples.

## 3. Results and Discussion

### 3.1. Comparison of XGBoost Performance Based on KPIC2 and XCMS

#### 3.1.1. Results of Feature Extraction

##### Liquor Dataset

**Figure 1:**
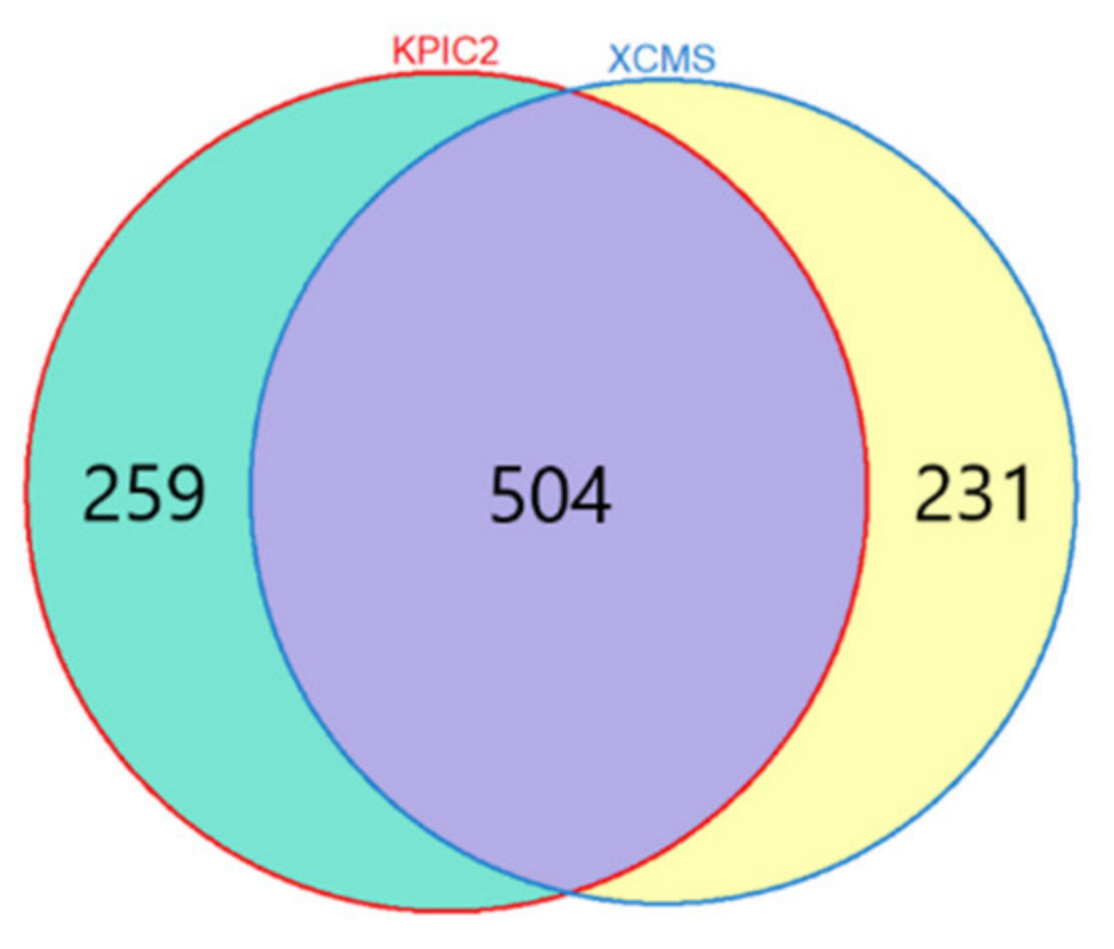
Venn diagram of the numbers of features in liquor dataset extracted by KPIC2 and XCMS. There are 259 features that are unique in KPIC2, and 231 features are also unique in XCMS. There are 504 features that are extracted by both KPIC2 and XCMS, which indicated the reliability of extraction results.

Features of the liquor samples were extracted by XCMS and KPIC2, and the numbers of extracted features are shown in the Venn diagram (Figure 1). The centWave method was used as a peak detection algorithm in XCMS, and the parameters of XCMS were optimized by the software package IPO. The parameters in KPIC2 were set to the same values if they have the same meaning as the parameters in XCMS.

As shown in Figure 1, the numbers of features by KPIC2 and XCMS were 763 and 735, respectively, and 504 common features were extracted by both methods. Therefore, approximately 75% of the features can be extracted by both KPIC2 and XCMS, which means that PICs detected by KPIC2 method are reliable. There are also some unique features for each method because of their different principles. A detailed description of the rationality of KPIC2 has been explained in the reference [29].

##### LCC Dataset

**Figure 2:**
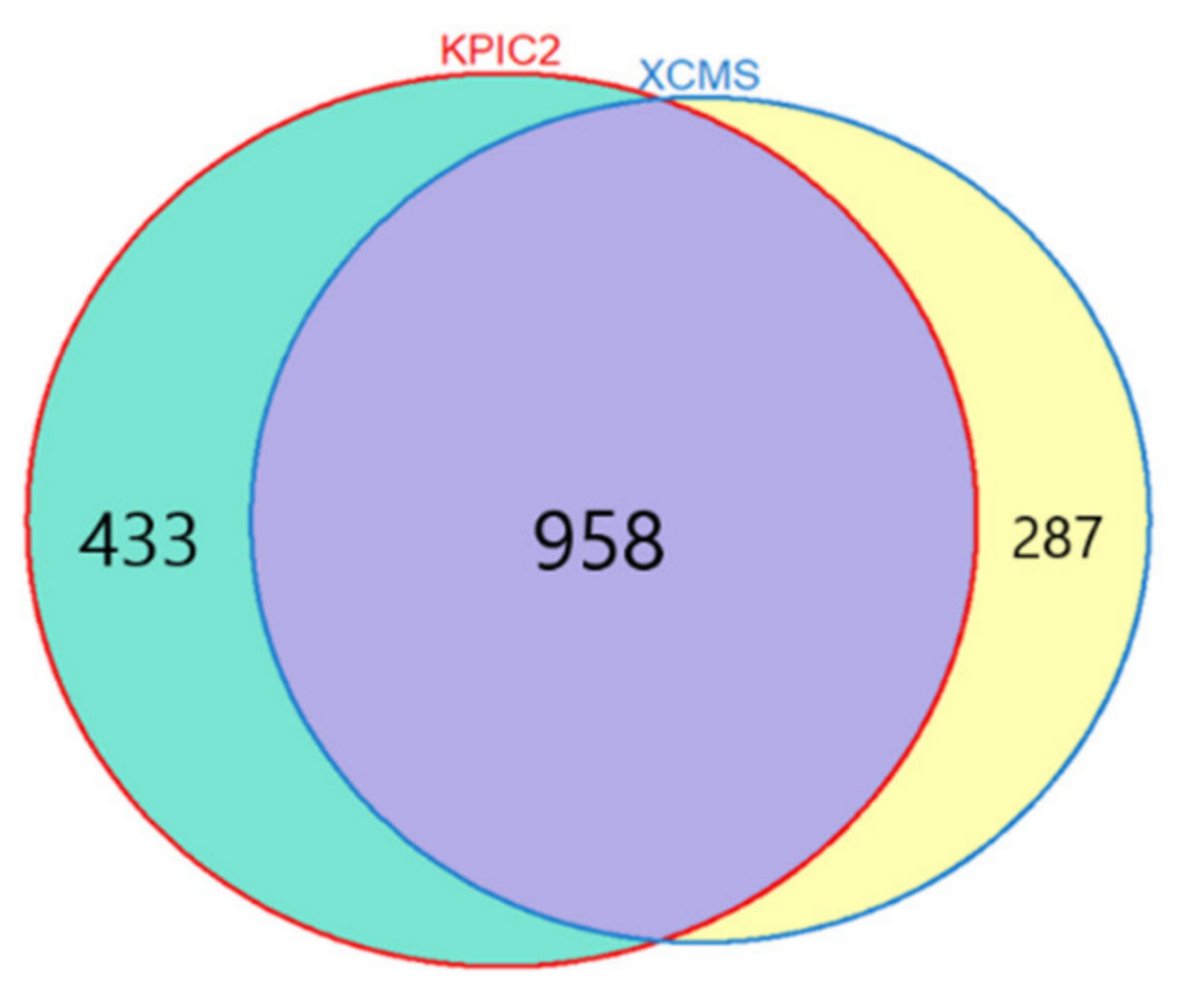
Venn diagram of the numbers of features in LCC dataset extracted by KPIC2 and XCMS. There are 433 features that are unique in KPIC2, and 287 features are also unique in XCMS. There are 958 common features detected by both KPIC2 and XCMS, which indicated the reliability of extraction results.

The LCC dataset was processed by KPIC2 and XCMS. Similarly, the parameters of XCMS are optimized by IPO. The parameters of KPIC2 were set according to XCMS. The numbers of extracted features are shown in Figure 2. It can be seen from the figure that approximately 70% of features can be detected by both KPIC2 and XCMS. The numbers of unique features by KPIC2 and XCMS were 433 and 287, respectively, and 958 common features were extracted by both methods. There are also some unique features for each method because of their different principles. There are more features extracted by KPIC2 than XCMS, which may be because of KPIC2 can eliminate noise signals and the true features are not covered by noise [29].

#### 3.1.2. Discriminant Models for Liquor and LCC Datasets

The XGBoost model is an advanced machine learning algorithm with fast calculation speed and excellent performance, and it is robust to overfitting. Therefore, XGBoost was used to build discriminant models based on the liquor and LCC datasets. The discriminant model was trained with the training set to learn decision rules for future prediction. Since XCMS is still widely used, it is necessary to study the performance of the XGBoost model based on KPIC2 compared with XCMS. In this study, the training sets of the liquor and LCC datasets were used for training the XGBoost models. With the established models, we can compare the discriminant ability of features extracted by XCMS and KPIC2.

The parameters of the XGBoost directly affect the performance of the model. The basis of the XGBoost algorithm is the gradient boosting algorithm, so the parameters (including n_estimators, learning_rate, silent and subsample) related to the boosting algorithm are first optimized. Then, the parameters (including max_depth, booster, gamma, min_child_weight and colsample_bytree) related to the weak learner are optimized through cross-validation. The optimal parameters were used to build the XGBoost model. The performance of the models was evaluated by the evaluation criteria in Section 2.6. The optimized parameters and evaluation criteria of the models are shown in Table 1. The results show that the XGBoost model on the test sets based on KPIC2 has high accuracy, precision, recall and F1 score. This means that the extracted features by KPIC2 have a higher discriminant ability. Therefore, the combination of KPIC2 and XGBoost is reasonable, which can more efficiently and accurately classify different sample groups. Moreover, this lays the foundation for the subsequent accurate analysis of differential metabolites.

The confusion matrices of XGBoost models on the test sets of the liquor and LCC datasets are shown in Table 2 and Table 3, respectively. From the results of the liquor dataset listed in Table 2, it can see that the KPIC2-based XGBoost model achieves 100% testing accuracy, while XCMS only has 92% testing accuracy. The result of classification in the LCC dataset is shown in Table 3, and the classification performance of the extraction results based on KPIC2 is better than that of XCMS. 

**Figure 3:**
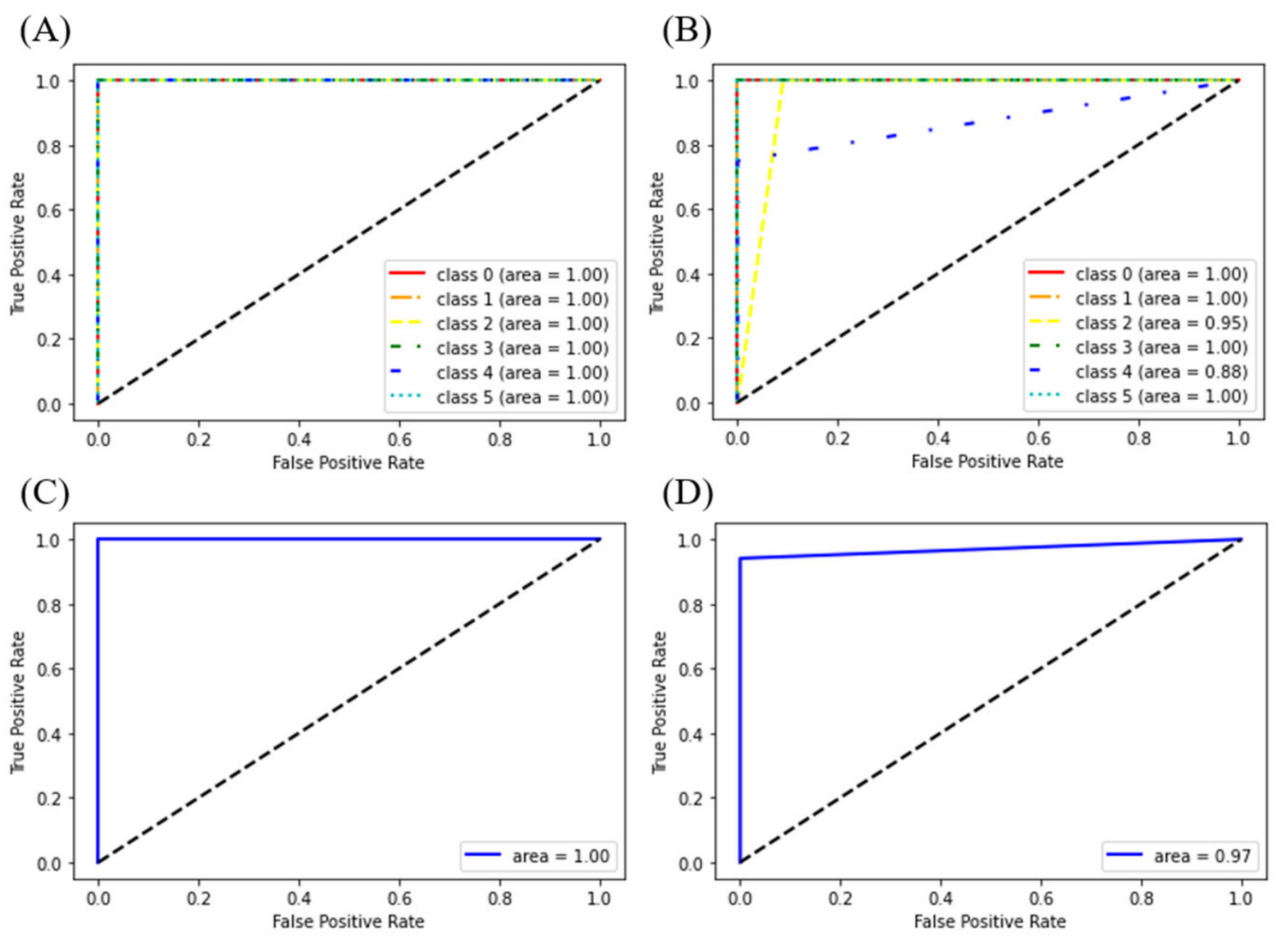
The receiver operating characteristic (ROC) curves of XGBoost models on the liquor dataset and the LCC dataset. Each color represents a class. (**A**) The ROC curve of XGBoost model trained by features of KPIC2 on the liquor dataset; (**B**) the ROC curve of XGBoost model trained by features of XCMS on the liquor dataset; (**C**) the ROC curve of XGBoost model trained by features of KPIC2 on the LCC dataset; (**D**) the ROC curve of XGBoost model trained by features of XCMS on the LCC dataset.

The trained model assigns a probability score to each peak group as a true peak. At the threshold cut-off value of each score, true positive rate and false positive rate were determined. The ROC curve was used to investigate the performance of the machine learning model at each threshold. In the liquor dataset, the ROC curves of the XGBoost model with features extracted by KPIC2 and XCMS are displayed in Figure 3A,B. The AUC of the test set of KPIC2 is 1.00, indicating that the model can better classify samples from different groups. Since it is multi-class problem, the true positive rate and false positive rate of each class under the threshold of each score are calculated and shown in Figure 3. In the LCC dataset, the AUC of each class is shown in Figure 3C,D, and the AUC value based on the results extracted by KPIC2 is better than the results of XCMS on the whole. 

### 3.2. Visualization of Liquor and LCC Datasets

Here, PCA, t-SNE and UMAP were used to reduce dimensionality and visualize samples in the liquor and LCC datasets. KPIC2 was used to extract the peak tables from these datasets. Then, the peak tables were visualized by PCA, t-SNE and UMAP. Since the data of real metabolomic samples often have some uncertainties, and we did not know ground-truth global and local structures. Therefore, we chose the Woolly Mammoth dataset to evaluate the differences between PCA, t-SNE and UMAP (Figures S1 and S2). It can be seen from the results that UMAP can achieve the best visualization results in the preservation of both local and global structures in the reduced dimensions compared to PCA and t-SNE.

**Figure 4:**
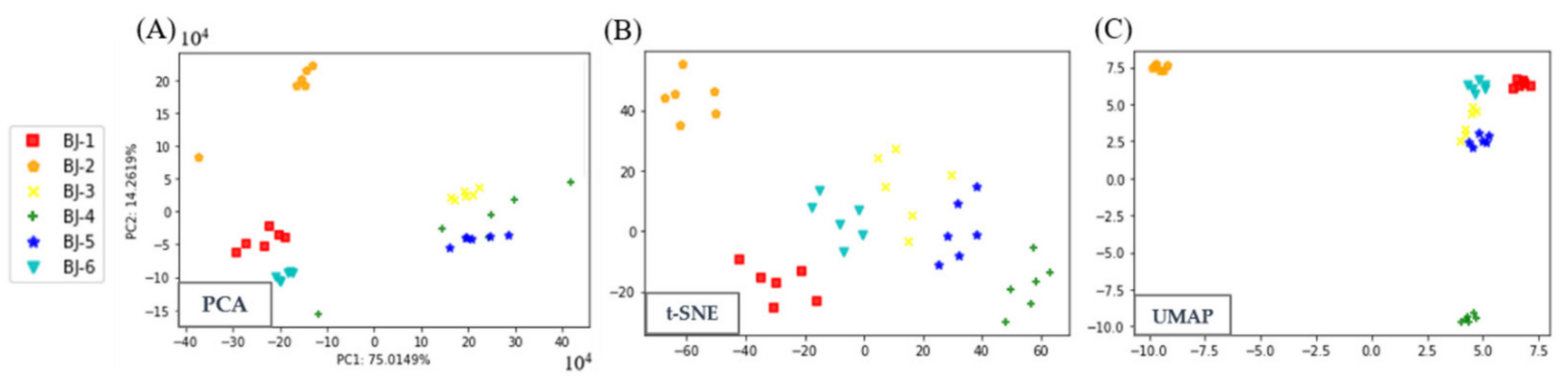
Visualization of the liquor dataset by PCA, t-SNE and UMAP. Each shape represents a sample. (**A**) The PCA plot based on features extracted by KPIC2 of the liquor dataset, and the percentage of variance explained by each selected component is displayed on the axis; (**B**) the t-SNE plot based on features extracted by KPIC2 of the liquor dataset; (**C**) the UMAP plot based on features extracted by KPIC2 of the liquor dataset.

In Figure 4, the PCA, t-SNE and UMAP analysis of the liquor dataset is shown. In Figure 4A, BJ-3, BJ-5 and BJ-6 are not well separated. Compared with the results of PCA, t-SNE can separate different liquor samples (Figure 4B). In Figure 4C, UMAP analysis can separate different liquor samples under a tighter coordinate axis, and the degree of aggregation within each group is closer. This is because it can keep both the global data structure and the local data structure by adjusting the parameter values of n_neighbors, and min_dist is used to adjust the tightness between different samples. Therefore, the separation trend of UMAP is better than PCA and t-SNE between different clusters of the liquor dataset.

**Figure 5:**
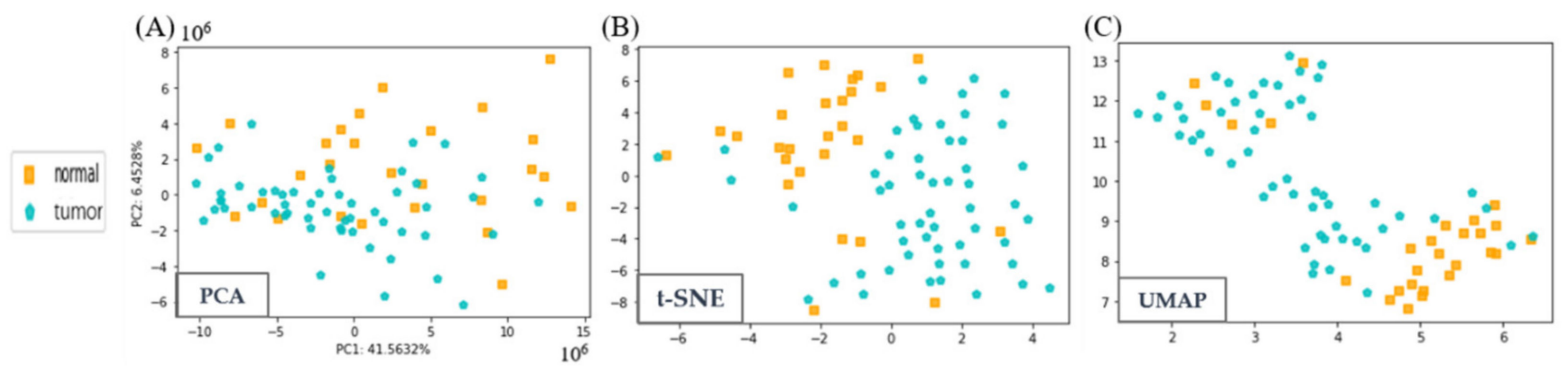
Visualization of the LCC dataset by PCA, t-SNE and UMAP. Each shape represents a sample. (**A**) The PCA plot is based on features extracted by KPIC2 of the LCC dataset, and the percentage of variance explained by each selected component is displayed on the axis; (**B**) the t-SNE plot is based on features extracted by KPIC2 of the LCC dataset; (**C**) the UMAP plot is based on features extracted by KPIC2 of the LCC dataset.

For the LCC dataset, the separation boundary between the two groups is not obvious, and the samples in the group are too scattered in the PCA plot (Figure 5A). Compared with the PCA plot, the t-SNE analysis makes the trend of separation between the two groups more obvious, but the group is still in a state of dispersion (Figure 5B). In Figure 5C, the two sets of samples also have a clear trend of separation, and UMAP can make the aggregation trend of samples better on a smaller axis. Therefore, UMAP can better show the aggregation tendency of the sample, and the tightness within the sample is smaller. This shows that the features extracted by KPIC2 lay the solid foundation for subsequent statistical analysis. The introduction of the UMAP method can better visualize the aggregation trend between different samples. Compared to PCA, UMAP is a non-linear dimensionality-reduction method, which has wider applicability to datasets.

### 3.3. Comparison of Classification Models Based on KPIC2

PLS-DA is a linear discriminant model based on PLS regression, which can be used for predictive and descriptive modeling as well as for discriminative variable selection. SVM can specify different kernel functions for decision-making functions. RF is an ensemble learning method that works by constructing multiple decision trees. Both SVM and RF are methods that can be applied to nonlinear modeling. Based on the feature detection results of KPIC2, different classification algorithms (PLS-DA, SVM, RF and XGBoost) were compared. The classification results are evaluated in terms of the accuracy, precision, recall and F1 value of performance measurement indicators of the machine learning model. The classification results of the two datasets are shown in Table 4.

It can be seen from Table 4 that the traditional linear discriminant model PLS-DA is obviously not applicable, and the five evaluation indicators of the model are significantly lower than other modeling results. SVM and RF can deal with nonlinear problems, which have a wider range of applications. The kernel type of the SVM model can be chosen, and adding a regular term can avoid overfitting. Therefore, the performance of SVM can sometimes be better classified compared to RF. It can be seen from Figure 4A and Figure 5A that samples of different groups do not have a good clustering trend even using the nonlinear dimensionality reduction methods. Therefore, these two datasets have a certain degree of non-linearity. This once again confirms the poor effect of the PLS-DA model. For the liquor dataset, the classification accuracy, precision, recall rate, specificity and F1 score of SVM are all higher than RF. The performance of the XGBoost model is much higher than that of PLS-DA, SVM and RF. This shows that the introduction of XGBoost can classify different samples well, and the accuracy of the discriminant model will be greatly improved. This may be because the XGBoost model uses the iteratively learning weak classifiers to reduce both bias and variance of the ensemble model and introduces the regular terms to avoid overfitting. For the LCC dataset, the classification performance of XGBoost model is still higher than PLS-DA, SVM and RF, and the classification results of the nonlinear discriminant models are better than PLS-DA. Among them, the classification performance of RF is better than SVM. This may be since that the data in the mapped space of SVM is not so linearly separable, which reduces the generalizability of the model, resulting in lower accuracy than RF. Therefore, the introduction of the XGBoost model can greatly improve the accuracy of data analysis, and it can be widely used in linear and non-linear datasets. In the future, the screening and identification of differential metabolites will become more accurate.

In addition, the quality of the model is crucial for accurately extracting differential metabolites. After the boosting tree in XGBoost is created, the importance score of each feature can be directly obtained. Therefore, the trained XGBoost model can automatically calculate feature importance, and the differential metabolites can be screened by feature importance of XGBoost. In the study, the higher accuracy of the model was obtained by the introduction of XGBoost, which will improve the accuracy of the differential metabolites screened.

## 4. Conclusions

In this study, we extended the KPIC2 with UMAP and XGBoost to analyze the complex LC–MS-based untargeted metabolomic datasets. KPIC2 was used to extract PICs from the liquor and LCC datasets. UMAP and XGBoost are used to visualize and discriminate complex samples, respectively. The performance of the XGBoost model based on the extraction results of KPIC2 is compared with XCMS. Results show that the features extracted by KPIC2 can achieve better classification accuracy (100%) on the test sets of the liquor dataset when compared with XCMS (92%). The result based on KPIC2 is also better than that of XCMS on the LCC dataset. Therefore, the combination of KPIC2 and XGBoost model is reasonable, and it can be used to classify features extracted based on KPIC2. By visualizing the Woolly Mammoth dataset, it can be shown that UMAP has the best visualization results in the preservation of both local and global structures in the reduced dimensions. The PCA, t-SNE and UMAP methods were used to visualize the liquor and LCC datasets, which also shows that UMAP can generate reasonable visualization in reduced dimensions for metabolomic datasets. Finally, the performance of the XGBoost model with features extracted by KPIC2 exceeds PLS-DA, SVM and RF on these two datasets. The combination of KPIC2, UMAP and XGBoost have the potential to be a promising pipeline to analyze LC–MS-based untargeted metabolomic datasets of complex samples. In addition, this method can effectively avoid overfitting due to the introduction of regular terms in the XGBoost model. In the future, we will use the feature importance of XGBoost to screen the biomarkers for further pathway analysis and mechanism investigations.

## Figures and Tables

**Table 1:** The parameters of optimized models and their evaluation criteria on the test sets.

		**KPIC2**		**XCMS/CAMERA**	
		**Liquor Dataset**	**LCC Dataset**	**Liquor Dataset**	**LCC Dataset**
Parameters	n_estimators	11	20	11	22
	learning_rate	0.01	0.01	0.1	0.01
	silent	1	1	1	1
	subsample	1	0.9	1	1
	max_depth	2	1	3	1
	booster	gbtree	gbtree	gbtree	gbtree
	gamma	0	0	0	0
	min_child_weight	1	3	1	1
	colsample_bytree	0.1	0.2	0.1	0.5
Model	Accuracy	1.000	1.000	0.917	0.963
	Precision	1.000	1.000	0.917	1.000
	Recall/Sensitivity	1.000	1.000	0.958	0.941
	Specificity	1.000	1.000	0.958	1.000
	F1_score	1.000	1.000	0.921	0.970

**Table 2:** Classification results of the liquor dataset on the test sets.

Actual		BJ-1	BJ-2	BJ-3	BJ-4	BJ-5	BJ-6	Class_Error	Error_Rate

KPIC2	BJ-1	1	0	0	0	0	0	0	0.00%
	BJ-2	0	2	0	0	0	0	0	
	BJ-3	0	0	1	0	0	0	0	
	BJ-4	0	0	0	2	0	0	0	
	BJ-5	0	0	0	0	4	0	0	
	BJ-6	0	0	0	0	0	2	0	
XCMS/ CAMERA	BJ-1	1	0	0	0	0	0	0	8.33%
	BJ-2	0	2	0	0	0	0	0	
	BJ-3	0	0	1	0	0	0	0	
	BJ-4	0	0	0	2	0	0	0	
	BJ-5	0	0	1	0	3	0	0.25	
	BJ-6	0	0	0	0	0	2	0	

**Table 3:** Classification results of the LCC dataset on the test sets.

Actual		Normal	Tumor	Class_Error	Error_Rate

KPIC2	Normal	10	0	0	0.00%
	Tumor	0	17	0	
XCMS/CAMERA	Normal	10	0	0	3.70%
	Tumor	1	16	0.06	

**Table 4:** Comparison of classification performance of different classification models on the test sets.

Dataset	Model	Accuracy	Precision	Recall	Specificity	F1_score
Liquor	PLS-DA	0.667	0.611	0.833	0.833	0.689
	SVM	0.833	0.889	0.917	0.917	0.861
	RF	0.750	0.875	0.875	0.875	0.780
	XGBoost	1.000	1.000	1.000	1.000	1.000
LCC	PLS-DA	0.741	0.778	0.824	0.600	0.800
	SVM	0.852	0.842	0.941	0.700	0.889
	RF	0.963	0.944	1.000	0.900	0.971
	XGBoost	1.000	1.000	1.000	1.000	1.000

## Data Availability

All raw data and associated metadata used in the LCC dataset are publicly available on the MetaboLights repository with identifier MTBLS1129 (https://www.ebi.ac.uk/metabolights/MTBLS1129, accessed date: 5 May 2021). The liquor dataset can be downloaded at: http://software.tobaccodb.org/software/antdas2 (accessed date: 5 May 2021).

## Supplementary Materials

The following are available online, Dataset: Introduction to the Woolly Mammoth dataset. Figure S1: Visualization of the Woolly Mammoth dataset by PCA, t-SNE and UMAP. Code: The code of features extraction methods (KPIC2), visualization methods (UMAP) and modeling methods (XGBoost).
