# Supplementary material for: Pure Ion Chromatograms Combined with Advanced Machine Learning Methods Improve Accuracy of Discriminant Models in LC–MS-Based Untargeted Metabolomics
Source: Molecules. 2021 May 5;26(9):2715. doi: 10.3390/molecules26092715 (PMC8125400; doi:10.3390/molecules26092715)
Supplement: Supplementary file 1 [file molecules-26-02715-s001.zip › molecules-1193374-supplementary.pdf]

molecules

Supplementary Materials

Pure Ion Chromatograms Combined with Advanced Machine Learning Methods Improve Accuracy of Discriminant Models in LC-MS-based Untargeted Metabolomics

Miao Tian[1], Zhonglong Lin[2], Xu Wang[3], Jing Yang[3], Wentao Zhao[3], Hongmei Lu[1], Zhimin Zhang[1,*], Yi Chen[2,*]

1. College of Chemistry and Chemical Engineering, Central South University, Changsha 410083, China. miaolcq@csu.edu.cn (M. T.); hongmeilu@csu.edu.cn (H. L.); zmzhang@csu.edu.cn (Z. Z.).
2. Yunnan Academy of Tobacco Agricultural Sciences, Kunming, Yunnan 650021, China. cytobacco007@sina.com (Y. C.); sdlzl1983@163.com (Z. L.).
3. Shanghai New Tobacco Product Research Institute Limited Company, Shanghai, 200082, China. wangx@sh.tobacco.com.cn (X. W.); yzc1985257@163.com (J. Y.); gj201323050@163.com (W. Z.).

* Correspondence: zmzhang@csu.edu.cn (Z.Z.); cytobacco007@sina.com (Y.C.)

Dataset. Introduction to Woolly Mammoth dataset

Woolly Mammoth dataset

The Woolly Mammoth dataset can be downloaded at https://github.com/MNoichl/UMAP-examples-mammoth-. As shown in Figure S1, the 3D scan of the Woolly Mammoth is shown.

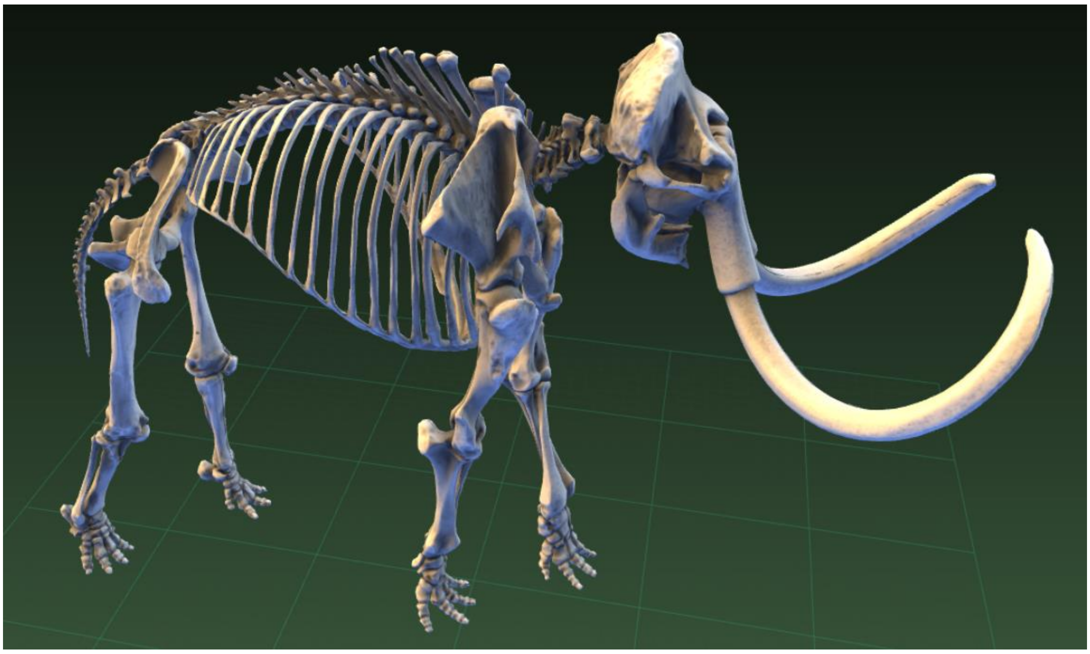

Figure S1. A 3D scan of the Wolly Mammoth in the Ice Age Hall of the National Museum of Natural History.

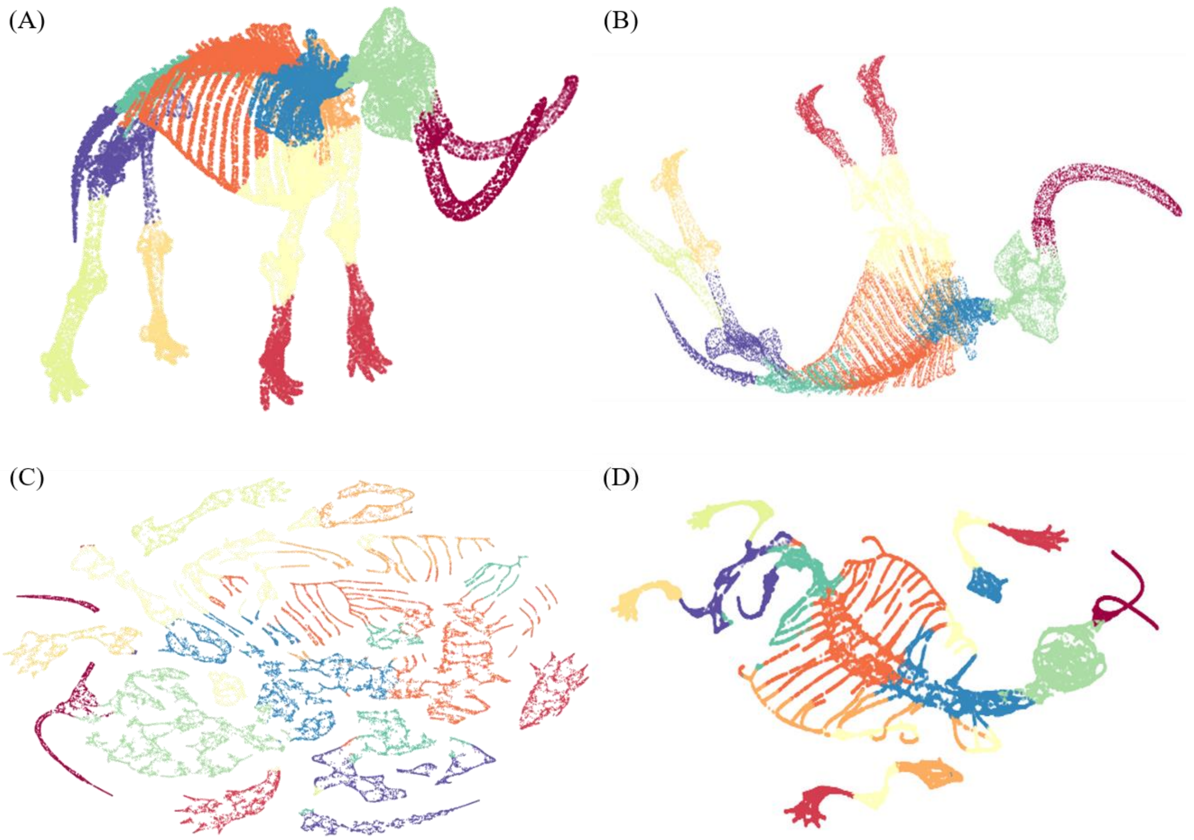

Figure S2. Visualization of Wolly Mammoth dataset by PCA, t-SNE and UMAP. (A) Original 3D plot of Woolly Mammoth dataset; (B) The PCA plot of the Wolly Mammoth dataset after dimensionality reduction in two-dimensional space; (C) The t-SNE plot of the Wolly Mammoth dataset after dimensionality reduction in two-dimensional space; (D) The UMAP plot of the Wolly Mammoth dataset after dimensionality reduction in two-dimensional space.

The Wolly Mammoth dataset is analyzed by PCA, t-SNE and UMAP. The results of the visual analysis of the Wolly Mammoth dataset are shown in Figure S2. In Figure S2 (A), the original 3D plot of the dataset is displayed, and it is used to evaluate the dimensionality reduction results of the three methods. It can be seen from the Figure S2 (B), the result of dimensionality reduction performed by PCA shows the global structure of the data, but the clustering trend within the dataset is not obvious. Therefore, PCA analysis can well display the global structure of the data while ignoring some local structures. In Figure S2 (C), the visualization results of the dataset are obtained by t-SNE. Although t-SNE makes the samples in the group have a better tendency to gather, it can't display the global structure of the data well compared with the PCA plot. For example, the ivory that belongs to the same kind has a tendency to separate. In Figure S2 (D), it can be seen that there is a balance between the global structure and the local structure of the data in the reduced dimensionalities by UMAP. It not only makes the aggregation trend of the samples better, but also preserves the overall structure of the data. This indicates that UMAP can achieve a balance between the local structure and the global structure of the data. In addition, the running speed of UMAP is much faster than that of t-SNE.

Code: The code of features extraction methods (KPIC2), visualization methods (UMAP) and modeling methods (XGBoost).

```
library(KPIC)
library(umap)
library(caret)
library(xgboost)

files <- 'Path'   # The path of the mzML or mzXML file
PICS <- PICset.kmeans(files, level=100, min_snr = 6, mztol = 0.05, gap = 3, width = c(5, 30), export=F, par=T, equal = TRUE)
PICS <- PICset.split(PICS)
PICS <- PICset.getPeaks(PICS)
groups_raw <- PICset.group(PICS, tolerance = c(0.01, 10))
groups_align <- PICset.align(groups_raw, method='fftcc', move='loess')
groups_align <- PICset.group(groups_align$picset, tolerance = c(0.01, 10))
groups_align <- PICset.align(groups_align, method='fftcc', move='direct')
groups_align <- groupCombine(groups_align, min_corr = 0.9, type='isotope', window = 10)
data <- getDataMatrix(groups_align)
data <- fillPeaks.EIBPC(data)
write.csv(data[["peakmat"]], file="Path")
write.csv(data[["data.mat"]], file="Path")
# UMAP plot
data <-   # Import data in "data frame" format
labels <-   # The label corresponding to the data is imported, format as "factor" type
umap = umap(data)
plot(umap[["layout"]][,1],.umap[["layout"]][,2], col = labels)
# Divide training set and test set
set.seed(1234)
index<-createDataPartition(labels, p=.67, list = FALSE)
train<-data[index,]
test<-data[-index,]
# After dividing the training set and the test set according to the labels, export the training data in csv format, the test data and their corresponding labels (train_data, test_data, train_label, test_label). Then, these csv files are re-imported.
# Data preprocessing
train_label <- as.numeric(train_label)
```

```
test_label<-as.numeric(test_label)
dtrain <- xgb.DMatrix(data = as.matrix(train_data), label=train_label)
dtest <- xgb.DMatrix(data = as.matrix(test_data), label=test_label)
# Training model
model <- xgboost(data = dtrain, max.depth = 2, eta = 1, nthread = 2, nrounds = 2)
pred <- predict(model, dtest)
print(model)
# Confusion matrix
pre_xgb = round(predict(model,newdata = dtest))
table(test_label,pre_xgb, dnn=c("true","pre"))
# Sort the importance of features.
importance_matrix <- xgb.importance(model = model)
print(importance_matrix)
xgb.plot.importance(importance_matrix = importance_matrix)
```
